# Association of Smoking, Comorbidity, Clinical Stage, and Treatment Intent With Socioeconomic Differences in Survival After Oropharyngeal Squamous Cell Carcinoma in Denmark

**DOI:** 10.1001/jamanetworkopen.2022.45510

**Published:** 2022-12-07

**Authors:** Maja Halgren Olsen, Kirsten Frederiksen, Pernille Lassen, Charlotte Rotbøl, Trille Kristina Kjaer, Jørgen Johansen, Hanne Primdahl, Elo Andersen, Claus Andrup Kristensen, Maria Andersen, Mohammad Farhadi, Jens Overgaard, Susanne Oksbjerg Dalton

**Affiliations:** 1 Department of Experimental Clinical Oncology, Aarhus University Hospital, Aarhus, Denmark; 2 Survivorship and Inequality in Cancer, Danish Cancer Society Research Center, Copenhagen, Denmark; 3 Statistics and Data Analysis, Danish Cancer Society Research Center, Copenhagen, Denmark; 4 Department of Oncology, Aalborg University Hospital, Aalborg, Denmark; 5 Danish Centre for Particle Therapy, Aarhus University Hospital, Aarhus, Denmark; 6 Department of Oncology, Odense University Hospital, Odense, Denmark; 7 Department of Oncology, Aarhus University Hospital, Aarhus, Denmark; 8 Department of Oncology, Herlev University Hospital, Herlev, Denmark; 9 Department of Oncology, Copenhagen University Hospital, Rigshospitalet, Copenhagen, Denmark; 10 Department of Clinical Oncology and Palliative Care, Zealand University Hospital, Næstved, Denmark

## Abstract

**Importance:**

The socioeconomic gap in survival after cancer is pronounced among patients with head and neck cancer. Understanding the mechanisms of this gap is crucial to target intervention strategies.

**Objective:**

To investigate socioeconomic differences in survival after oropharyngeal squamous cell carcinoma (OPSCC) according to human papillomavirus (HPV) status and the extent to which smoking, comorbidity, clinical stage, and treatment intent explain the survival gap.

**Design, Setting, and Participants:**

This nationwide, population-based cohort study was based on prospectively collected information on all patients with a diagnosis of OPSCC from the Danish Head and Neck Cancer Group database and administrative registries. The study included 4600 patients born in 1921 or later, aged 30 years or older, and residing in Denmark 1 year prior to OPSCC diagnosis. Patients with missing information (547 [12%]) were excluded. Patients were diagnosed between January 1, 2008, and December 31, 2019, and followed up until December 31, 2021. Data were analyzed from June 6 to October 4, 2022.

**Exposure:**

Socioeconomic position (educational level, disposable income, or cohabiting status).

**Main Outcomes and Measures:**

Socioeconomic differences in 5-year overall survival were estimated in Cox proportional hazards regression models by HPV status. The indirect effect and proportion mediated by smoking, comorbidity, clinical stage, and treatment intent were estimated based on a counterfactual approach.

**Results:**

The analyzed cohort comprised 4053 patients (1045 women [26%] and 3008 men [74%]). The median age was 61 years (IQR, 55-68 years), and 2563 patients (63%) had HPV-positive OPSCC while 1490 patients (37%) had HPV-negative OPSCC. The 5-year standardized overall survival was 10% to 15% lower among patients with a lower educational level, with low disposable income, or who were living alone (patients with HPV-positive OPSCC, 68%-71%; patients with HPV-negative OPSCC, 31%-34%) than patients with a higher educational level, high disposable income, or a cohabiting partner (patients with HPV-positive OPSCC, 81%-86%; patients with HPV-negative OPSCC, 43%-46%). Among patients with HPV-positive OPSCC, a considerable part of this survival gap was estimated to be associated with differences in smoking (27%-48%), comorbidity (10%-19%), clinical stage (8%-19%), and treatment intent (16%-28%). Among those with HPV-negative OPSCC, comorbidity (12%-22%) and treatment intent (16%-42%) were the primary potential mediators.

**Conclusions and Relevance:**

This cohort study suggests that, regardless of HPV status, patients with low socioeconomic position had 10% to 15% lower 5-year overall survival than patients with high socioeconomic position. A substantial part of this survival gap was associated with differences in smoking, comorbidity, clinical stage, or treatment intent at diagnosis.

## Key Points

**Question:**

Is the socioeconomic gap in survival after oropharyngeal squamous cell carcinoma (OPSCC) associated with differences in smoking, comorbidity, clinical stage, and treatment intent?

**Findings:**

In a nationwide, population-based cohort study of 4053 patients with a diagnosis of human papillomavirus (HPV)–positive or HPV-negative OPSCC, a considerable part of the observed socioeconomic survival gap was associated with differences in smoking status, comorbidity, clinical stage, or treatment intent.

**Meaning:**

The results suggest the need for structural primary prevention initiatives targeting socioeconomic differences in health behavior and for tools that can identify vulnerable patients to support health promotion and best possible care for this group.

## Teaser

This cohort study investigates socioeconomic differences in survival after oropharyngeal squamous cell carcinoma according to human papillomavirus status and the extent to which smoking, comorbidity, clinical stage, and treatment intent explain the survival gap.

## Introduction

The socioeconomic differences in cancer survival are pronounced for patients with a diagnosis of head and neck squamous cell carcinoma (HNSCC).^1,2,3^ In Denmark, patients with HNSCC with low income had 22% lower 5-year relative survival than patients with high income.^3^ Understanding where in the trajectory of HNSCC this socioeconomic gap arises is crucial to targeting intervention strategies.^4^

Socioeconomic position (SEP) is associated with health during the entire course of life and is shaped by differences in multiple contextual and individual factors (eg, health literacy, lifestyle, capability, use of the health care system, and communication with health care professionals).^4,5,6,7,8^ Socioeconomic differences in survival after cancer and, specifically, HNSCC have been suggested to be associated with factors related to disease stage at diagnosis, other disease characteristics, health behaviors, presence of other chronic conditions, and treatment.^9,10,11^ Particularly for HNSCC, differences in etiology may play an important role. A growing socioeconomic disparity in survival for patients with a diagnosis of HNSCC^1,2,3^ is paralleled by an increased number and proportion of human papillomavirus (HPV)–related cases of HNSCC, particularly oropharyngeal squamous cell carcinoma (OPSCC).^12^ Patients with HPV-positive OPSCC tend to have a higher SEP, smoke less, and have fewer comorbidities than patients with HPV-negative OPSCC.^13,14,15,16^ Human papillomavirus–positive OPSCC is also more sensitive to (chemo)radiotherapy, and survival is better compared with the HPV-negative disease.^16,17,18,19^ As a consequence, OPSCC has been classified according to HPV status.^20^

Most^14,15,21,22,23,24^ previous studies of the association between SEP and survival after HPV-positive or HPV-negative OPSCC^13,14,15,21,22,23,24^ have provided estimates adjusted simultaneously for multiple socioeconomic and intermediating factors. This adjustment likely underestimates the association between SEP and survival and bars interpretation of possible pathways leading to the observed inequalities. In a unique nationwide cohort of all Danish patients with a diagnosis of HPV-positive or HPV-negative OPSCC and treated according to national standardized guidelines, we examined the association between SEP and 5-year overall survival. Furthermore, we investigated the extent to which the observed socioeconomic gap in survival could be associated with differences in smoking status, comorbidity, clinical stage, and treatment intent.

## Methods

### Study Design and Setting

This nationwide, population-based cohort study was based on prospectively collected registry data. In Denmark, a social welfare system provides free tuition from primary to higher education, along with tax-funded health care services, largely free of copayments.^25^ Since 1968, a unique personal identification number has been assigned to all residents, enabling linkage between clinical databases and administrative registries.^25^ Since 1992, all patients with HNSCC treated in Denmark have been registered in the national clinical Danish Head and Neck Cancer Group (DAHANCA) database with information on, for example, date of diagnosis (date of first appointment at the oncologic center), cancer subsite, clinical stage, smoking, treatment intent, and—more recently—HPV-associated p16 expression.^12^ Treatment of OPSCC is standardized across all 6 oncologic centers by national clinical guidelines and has been uniform since 2008, with (chemo)radiotherapy as the primary modality.^26,27^ Statistics Denmark administers various yearly updated nationwide socioeconomic registers with individual-level information.^28,29^ The Central Population Register contains information on date of birth, legal gender, address, migration status, and vital status on all citizens residing in Denmark.^30^ The Danish National Patient Register records information on hospitalizations and outpatient visits.^31^ In agreement with the General Data Protection Regulation, this study is registered in the Danish Cancer Society’s internal project register database. According to Danish legislation, a register-based study with no contact with individuals does not require informed consent or ethical board review. This study followed the Strengthening the Reporting of Observational Studies in Epidemiology (STROBE) reporting guideline for observational studies.

### Study Population

We identified 4671 patients with a diagnosis of OPSCC (*International Statistical Classification of Diseases and Related Health Problems, Tenth Revision* codes C01, C05.1-9, C09, C10.0, and C10.2-9) during the period from January 1, 2008, through December 31, 2019. Included patients (n = 4600) were born in 1921 or later, 30 years of age or older at the date of diagnosis, and residing in Denmark in the year leading up to their diagnosis. Information on educational level was not available for patients born before 1921, and patients younger than 30 years were considered not to have established their SEP yet. Patients were followed up until the date of death; migration; December 31, 2021; or 5 years after diagnosis, whichever came first. The main analyses were based on complete cases (n = 4053), excluding patients with missing information on 1 or more of the included parameters (n = 547 [12%]) (eFigure 1 in Supplement 1). The analyzed cohort was divided into patients with HPV-positive OPSCC (n = 2563) and patients with HPV-negative OPSCC (n = 1490). Human papillomavirus association was determined by p16 immunohistochemistry staining, an established surrogate for tumor HPV in OPSCC.^17^ Tumors were classified as HPV positive in the case of strong and diffuse nuclear or cytoplasmatic staining in more than 70% of tumor cells.^17^

### SEP and Mediators

To analyze different aspects of SEP, we included and analyzed separately 3 different proxy indicators of SEP: educational level,^28^ personal disposable income,^32^ and cohabitation status.^33^ When similar results were observed, low SEP denoted a lower educational level, low disposable income, or living alone; medium SEP denoted a medium educational level or disposable income; and high SEP denoted a higher educational level, high disposable income, or living with a partner. Patients’ SEP was hypothesized to be associated with survival after HPV-positive and HPV-negative OPSCC through multiple pathways (eFigure 2 in Supplement 1). We analyzed 4 mediators: smoking status, comorbidity, clinical stage, and treatment intent at the time of diagnosis, which have previously been suggested as key factors (mediators) in the association between SEP and survival after cancer.^9^ Smoking was defined as current vs former or never smoker.^34^ Comorbidity was defined as no vs prior hospitalization or outpatient visit^31^ with a disease included in the revised Charlson Comorbidity Index for Head and Neck Cancer (HN-CCI).^35^ Clinical stage was defined according to the American Joint Committee on Cancer/Union for International Cancer Control (AJCC/UICC) TNM classification system (8th edition)^20^ as advanced (TNM-8: III-IV) vs early (TNM-8: I-II). As TNM-8 was introduced after 2017, a subanalysis included stage based on the 7th edition (TNM-7).^36^ Treatment intent was defined as curative intent vs palliative or no treatment.^26^ A detailed definition of variables is provided in eTable 1 in Supplement 1.

### Statistical Analysis

Data were analyzed from June 6 to October 4, 2022. Initially, crude overall survival probabilities within the first 5 years after diagnosis were calculated and illustrated in Kaplan-Meier curves by the 3 SEP indicators (educational level, disposable income, and cohabitation status) and 4 mediators (smoking, comorbidity, clinical stage, and treatment intent). Associations between each SEP indicator and mediator were expressed as odds ratios with 95% CIs as estimated in logistic regression models adjusted for gender, age, and calendar year at diagnosis.

The marginal association between each SEP indicator and 5-year all-cause mortality was estimated in multivariable Cox proportional hazards regression analyses adjusted for gender, age, and calendar year at diagnosis (both continuous). Based on the Cox proportional hazards regression models, we reported the standardized absolute 5-year survival probabilities and differences thereof according to SEP.^37,38^

To quantify the extent to which the association between each SEP indicator and 5-year survival was explained by the pathways through each of the 4 mediators separately and in combination, the total associations were decomposed into direct effects not associated with the mediator and indirect effects associated with the mediator.^39^ Estimation of direct and indirect effects was performed using a mediation formula approach (eMethods in Supplement 1).^40^ The proportion mediated was calculated as the indirect effect/(indirect effect + direct effect). The 95% CIs for direct and indirect effects were based on 1000 bootstrap samples. In sensitivity analyses, we included an exposure-mediator interaction term in the mediator-adjusted models. To validate the complete-case approach, we further investigated associations, applying different values to the missing values. All *P* values were from 2-sided tests and results were deemed statistically significant at *P* < .05. The analyses were performed in SAS, version 9.4 (SAS Institute Inc).

## Results

### Cohort Description

The analyzed cohort included 4053 patients (1045 women [26%] and 3008 men [74%]). The median age was 61 years (IQR, 55-68 years) (Table 1; eTable 2 in Supplement 1). In all, 2563 patients (63%) had HPV-positive disease, and 1490 (37%) had HPV-negative disease (Table 1). Within the 5-year period, 1425 patients died (Table 2), and 8 patients were censored owing to migration. The overall 5-year survival probability was 63% (95% CI, 61%-64%) and was considerably higher among patients with HPV-positive OPSCC (78% [95% CI, 76%-80%]) than among patients with HPV-negative OPSCC (37% [95% CI, 34%-39%]). A larger proportion of patients with HPV-positive OPSCC than patients with HPV-negative OPSCC had a higher educational level (651 of 2563 [25%] vs 209 of 1490 [14%]), high disposable income (1036 of 2563 [40%] vs 288 of 1490 [19%]), or a cohabiting partner (1739 of 2563 [68%] vs 678 of 1490 [46%]) (Table 1). Considering the mediators, a larger proportion of patients with HPV-positive OPSCC than patients with HPV-negative OPSCC were former or never smokers (1983 of 2563 [77%] vs 459 of 1490 [31%]), had no comorbid disease according to the HN-CCI (2123 of 2563 [83%] vs 941 of 1490 [63%]), were diagnosed at an early disease stage (TNM-8, I-II; 2262 of 2563 [88%] vs TNM-8, III-IV; 319 of 1490 [21%]), or were treated with curative intent (2460 of 2563 [96%] vs 1242 of 1490 [83%]).

**Table 1.** Patient Characteristics by HPV Status

Characteristic	Patients, No. (%)	
	HPV-positive OPSCC (n = 2563)	HPV-negative OPSCC (n = 1490)
Age, y		
30-53	585 (23)	200 (13)
54-58	520 (20)	261 (18)
59-63	519 (20)	307 (21)
64-68	441 (17)	319 (21)
69-95	498 (19)	403 (27)
Median (IQR)	60 (54-67)	63 (57-69)
Gender		
Male	1965 (77)	1043 (70)
Female	598 (23)	447 (30)
Year of diagnosis		
2008-2010	409 (16)	290 (19)
2011-2013	621 (24)	391 (26)
2014-2016	720 (28)	417 (28)
2017-2019	813 (32)	392 (26)
Educational level		
Lower	476 (19)	381 (26)
Medium	1436 (56)	900 (60)
Higher	651 (25)	209 (14)
Disposable income		
Low	477 (19)	525 (35)
Medium	1050 (41)	677 (45)
High	1036 (40)	288 (19)
Cohabitation status		
Living alone	824 (32)	812 (55)
Cohabiting	1739 (68)	678 (46)
Smoking status		
Current smoker	580 (23)	1031 (69)
Former smoker	1201 (47)	418 (28)
Never smoker	782 (31)	41 (3)
Comorbidities according to HN-CCI		
≥1	440 (17)	549 (37)
0	2123 (83)	941 (63)
Stage (TNM-8)		
Advanced	301 (12)	1171 (79)
Early	2262 (88)	319 (21)
Treatment intent		
Palliative or no treatment	103 (4)	248 (17)
Curative	2460 (96)	1242 (83)

Abbreviations: HN-CCI, Revised Charlson Comorbidity Index for Head and Neck Cancer; HPV, human papillomavirus; OPSCC, oropharyngeal squamous cell carcinoma; TNM-8, American Joint Committee on Cancer/Union for International Cancer Control TNM classification system, 8th edition.

**Table 2.** Five-Year Overall Survival, by HPV Status and Socioeconomic Indicator

Indicator	Patients with HPV-positive OPSCC					Patients with HPV-negative OPSCC				
	No.		% (95% CI)			No.		% (95% CI)		
	Death	Person-years	Crude^a^	Standardized^b^	Difference^c^	Death	Person-years	Crude^a^	Standardized^b^	Difference^c^
Educational level										
Lower	130	1775	70.7 (66.1 to 74.8)	70.8 (66.9 to 75.0)	−14.9 (−19.8 to −10.0)	250	953	31.9 (27.0 to 36.8)	31.4 (27.2 to 36.2)	−14.6 (−22.4 to −6.8)
Medium	303	5696	77.3 (74.9 to 79.5)	77.1 (75.0 to 79.4)	−8.6 (−12.1 to −5.1)	546	2437	36.7 (33.4 to 40.0)	37.1 (34.0 to 40.3)	−9.0 (−16.2 to −1.8)
Higher	85	2737	85.2 (82.0 to 87.9)	85.7 (83.0 to 88.5)	0 [Reference]	111	625	44.7 (37.6 to 51.6)	46.0 (39.9 to 53.1)	0 [Reference]
Disposable income										
Low	136	1779	69.4 (64.8 to 73.5)	70.2 (66.3 to 74.4)	−11.2 (−15.9 to −6.5)	330	1399	35.3 (31.1 to 39.5)	33.6 (29.8 to 37.8)	−9.7 (−16.3 to −3.0)
Medium	209	4184	78.4 (75.7 to 80.9)	78.6 (76.2 to 81.2)	−2.8 (−6.3 to 0.6)	408	1770	36.8 (32.9 to 40.6)	36.6 (33.2 to 40.4)	−6.6 (−13.0 to −0.3)
High	173	4246	81.7 (79.0 to 84.0)	81.4 (79.0 to 83.9)	0 [Reference]	169	846	38.8 (32.9 to 44.6)	43.3 (38.1 to 49.1)	0 [Reference]
Cohabitation status										
Living alone	250	3012	66.8 (63.2 to 70.1)	68.4 (65.3 to 71.7)	−14.5 (−18.2 to −10.8)	529	2031	32.4 (29.0 to 35.8)	31.5 (28.5 to 34.8)	−11.8 (−16.4 to −7.1)
Cohabiting	268	7196	83.3 (81.4 to 85.1)	82.9 (81.1 to 84.8)	0 [Reference]	378	1984	41.7 (37.8 to 45.5)	43.3 (39.7 to 47.1)	0 [Reference]

Abbreviations: HPV, human papillomavirus; OPSCC, oropharyngeal squamous cell carcinoma.

^a^ Kaplan-Meier estimates in strata by educational level, disposable income, and cohabitation status.

^b^ Five-year survival estimates standardized to gender, age (continuous), and calendar year (continuous) at diagnosis.

^c^ Difference in standardized 5-year survival estimates.

### SEP and Survival

**Figure 1.**
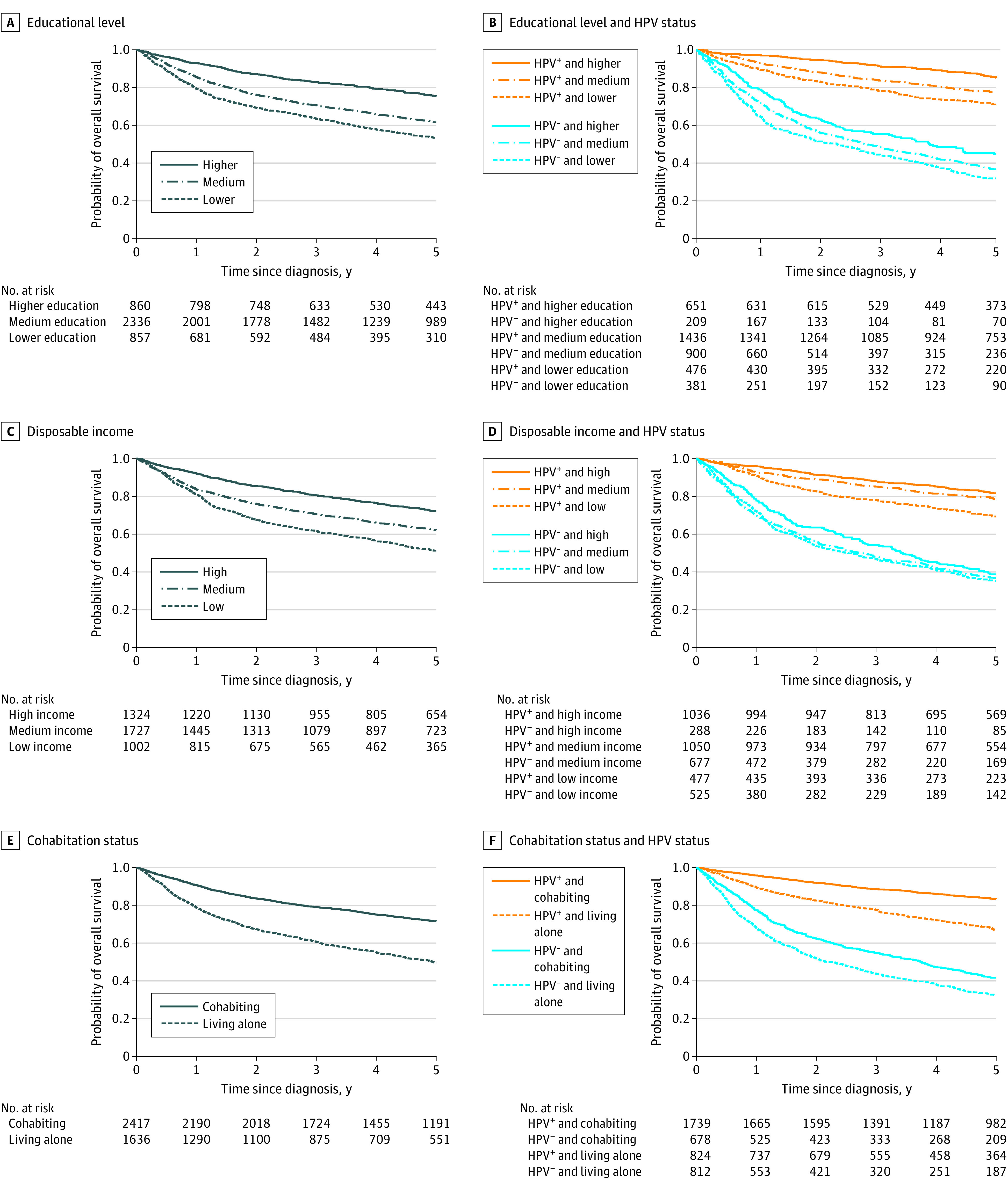
Overall Crude Survival Probability by Socioeconomic Indicator and Human Papillomavirus (HPV) Status A, Educational level. B, Educational level and HPV status. C, Disposable income. D, Disposable income and HPV status. E, Cohabitation status. F, Cohabitation status and HPV status. HPV^+^ indicates HPV positive; and HPV^−^, HPV negative.

Survival decreased gradually by decreasing SEP indicator among both patients with HPV-positive OPSCC and patients with HPV-negative OPSCC (Figure 1). Taking gender, age, and calendar year into account, the standardized 5-year overall survival among patients with HPV-positive OPSCC was 71% (95% CI, 67%-75%) among patients with a lower educational level, 70% (95% CI, 66%-74%) among patients with low disposable income, and 68% (95% CI, 65%-72%) among patients living alone. In contrast, the 5-year overall survival among patients with HPV-positive OPSCC was 86% (95% CI, 83%-89%) among patients with a higher educational level, 81% (95% CI, 79%-84%) among patients with high disposable income, and 83% (95% CI, 81%-85%) among patients with a cohabiting partner (Table 2). Even though the corresponding survival probabilities were considerably lower for patients with HPV-negative OPSCC (low SEP, 31% [95% CI, 27%-36%] to 34% [95% CI, 30%-38%]; high SEP, 43% [95% CI, 38%-49%] to 46% [95% CI, 40%-53%]) than for patients with HPV-positive OPSCC, the difference in 5-year standardized survival between patients with low and patients with high SEP was similar for patients with HPV-positive OPSCC (−11% [95% CI, –16% to –7%] to −15% [95% CI, –20% to –10%]) and patients with HPV-negative OPSCC (−10% [95% CI, –16% to –3%] to −15% [95% CI, –22% to –7%]).

### Associations Between SEP and Mediators

**Figure 2.**
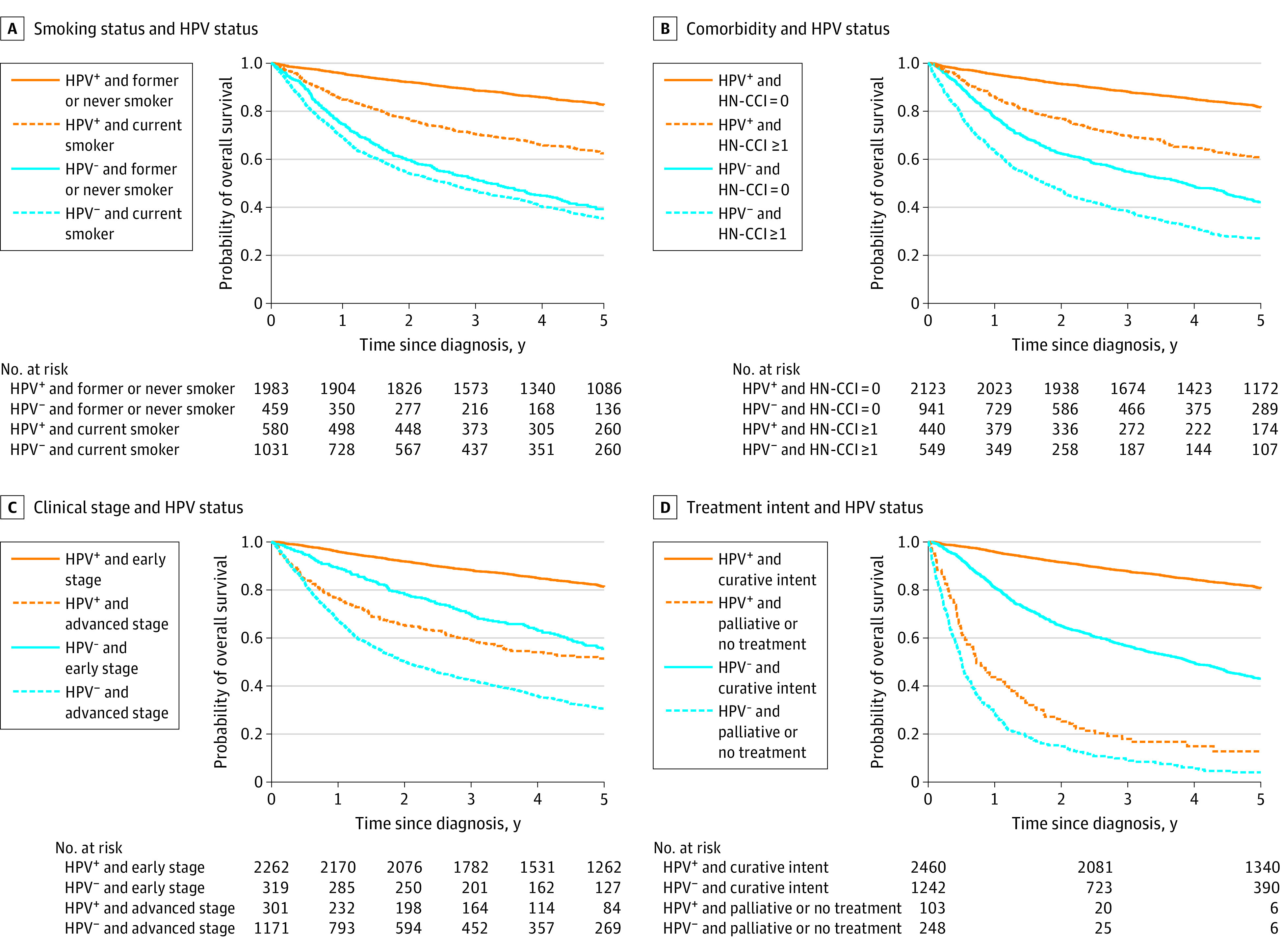
Overall Crude Survival Probability by Mediators and Human Papillomavirus (HPV) Status A, Smoking status and HPV status. B, Comorbidity and HPV status. C, Clinical stage and HPV status. D, Treatment intent and HPV status. HN-CCI indicates revised Charlson Comorbidity Index for Head and Neck Cancer; HPV^+^, HPV positive; and HPV^−^, HPV negative.

When considering the mediators, patients with low SEP had higher odds than patients with high SEP for being a current smoker, having 1 or more comorbid conditions according to the HN-CCI, having an advanced disease stage at diagnosis, or not receiving curative treatment. As an exception, we found no consistent associations between SEP and clinical stage (TNM-8) at diagnosis among patients with HPV-negative OPSCC (eTable 3 in Supplement 1) and neither among patients with HPV-positive OPSCC or those with HPV-negative OPSCC in subanalyses when using TNM-7 (eTable 4 in Supplement 1). All 4 mediators were associated with survival (Figure 2).

### Direct and Indirect Effects

Table 3 summarizes the mediation analyses, and hazard ratio estimates from the Cox proportional hazards regression models are provided in eFigure 3 in Supplement 1. For instance, among patients with HPV-positive OPSCC, the observed decreased 5-year survival among patients with a lower educational level compared with a higher educational level was decomposed into an estimated indirect effect of −4.7% (95% CI, −6.3% to −3.3%) that could be explained by differences in smoking status and a direct effect of −10.8% (95% CI, −15.4% to −6.1%) associated with other pathways (Table 3). This means that an estimated 30% of the observed difference in 5-year survival between patients with a lower education level and patients with a higher educational level was associated with differences in smoking status. Among patients with HPV-negative OPSCC, the indirect effect of smoking was weaker (−0.8% [95% CI, −1.7% to −0.1%]), corresponding to a proportion mediated of 5%. The proportion mediated by comorbidity was similar among patients with HPV-positive OPSCC and patients with HPV-negative OPSCC but varied across SEP indicators (10%-22%). The indirect effect of disease stage at diagnosis was statistically significant only among patients with HPV-positive OPSCC, corresponding to a proportion mediated of 8% to 19% across SEP indicators. Finally, the indirect effect of treatment intent varied according to HPV status and SEP indicator, with a proportion mediated of 16% to 42%. In combination, all 4 mediators explained 48% to 75% of the socioeconomic survival gap among patients with HPV-positive OPSCC. This is lower than the sum of the individual associations (smoking [27%-48%], comorbidity [10%-19%], clinical stage [8%-19%], and treatment intent [16%-28%]), indicating interaction between these mediators. For patients with HPV-negative OPSCC, all 4 mediators explained in combination 24% to 60% of the observed socioeconomic differences in survival (with comorbidity [12%-22%] and treatment intent [16%-42%] as primary potential mediators). The indirect effect of stage using TNM-7 was not statistically significant among patients with HPV-positive OPSCC (−0.1% [95% CI, −0.4% to 0.2%]) or patients with HPV-negative OPSCC (−1.3% [95% CI, −3.3% to 0.7%]). We obtained comparable results in models including an exposure-mediator interaction (eTable 5 in Supplement 1). Sensitivity analyses revealed no consistent differences in survival in associations from the complete cohort (eTable 6 in Supplement 1).

**Table 3.** Estimated Direct and Indirect Effects, by HPV Status and Socioeconomic Indicator

Indicator	Patients with HPV-positive OPSCC			Patients with HPV-negative OPSCC		
	Survival difference, % (95% CI)		Proportion mediated, %^a^	Survival difference, % (95% CI)		Proportion mediated, %^a^
	Direct effect^b^	Indirect effect^b^		Direct effect^b^	Indirect effect^b^	
Educational level (lower vs higher)						
Smoking status	−10.8 (−15.4 to −6.1)	−4.7 (−6.3 to −3.3)	30.1	−13.9 (−21.6 to −6.2)	−0.8 (−1.7 to −0.1)	5.4
Comorbidity	−13.0 (−17.4 to −8.3)	−2.4 (−3.7 to −1.4)	15.4	−12.9 (−20.8 to −5.2)	−1.9 (−3.5 to −0.6)	13.1
Clinical stage (TNM-8)	−12.5 (−17.5 to −7.5)	−2.0 (−3.5 to −0.6)	13.6	−13.4 (−20.9 to −6.1)	−1.3 (−3.3 to 0.6)	8.6
Treatment intent	−10.1 (−14.8 to −5.1)	−3.9 (−5.6 to −2.4)	28.1	−7.9 (−15.0 to −1.2)	−3.3 (−5.6 to −1.0)	29.2
Combined	−5.6 (−10.3 to −1.1)	−9.1 (−11.4 to −6.7)	62.1	−6.2 (−13.5 to −0.1)	−5.8 (−8.8 to −2.8)	48.2
Disposable income (low vs high)						
Smoking status	−6.3 (−10.8 to −2.5)	−5.8 (−7.6 to −4.1)	47.8	−9.1 (−15.7 to −2.5)	−0.5 (−1.3 to 0.0)	5.6
Comorbidity	−9.2 (−13.7 to −5.0)	−2.2 (−3.4 to −1.3)	19.2	−7.6 (−14.0 to −1.6)	−2.2 (−3.5 to −1.0)	22.1
Clinical stage (TNM-8)	−9.4 (−14.0 to −4.9)	−2.2 (−3.6 to −0.8)	18.7	−10.5 (−16.9 to −4.6)	0.3 (−1.5 to 2.1)	NA^c^
Treatment intent	−6.9 (−11.8 to −2.5)	−2.3 (−3.8 to −0.8)	24.8	−8.6 (−14.9 to −2.4)	−1.7 (−3.5 to 0.3)	16.2
Combined	−2.9 (−6.8 to 0.2)	−8.7 (−11.1 to −6.1)	74.9	−7.8 (−13.9 to −1.7)	−2.4 (−5.2 to 0.5)	23.8
Cohabitation status (living alone vs cohabiting)						
Smoking status	−10.6 (−14.1 to −7.0)	−3.9 (−5.2 to −2.8)	27.0	−11.0 (−15.7 to −6.6)	−0.8 (−1.6 to −0.2)	6.9
Comorbidity	−13.2 (−16.6 to −9.4)	−1.4 (−2.4 to −0.7)	9.9	−10.1 (−14.3 to −5.2)	−1.3 (−2.4 to −0.6)	11.7
Clinical stage (TNM-8)	−12.9 (−16.7 to −9.3)	−1.1 (−2.1 to −0.1)	7.7	−10.6 (−14.9 to −5.8)	−0.6 (−1.9 to 0.6)	5.3
Treatment intent	−10.8 (−14.4 to −7.0)	−2.1 (−3.3 to −1.1)	16.3	−5.6 (−9.9 to −1.2)	−4.1 (−5.6 to −2.7)	42.0
Combined	−6.7 (−10.2 to −3.6)	−6.2 (−8.0 to −4.3)	48.1	−3.9 (−9.2 to 0.4)	−5.9 (−8.1 to −3.8)	60.2

Abbreviations: HPV, human papillomavirus; NA, not applied; OPSCC, oropharyngeal squamous cell carcinoma; TNM-8, American Joint Committee on Cancer/Union for International Cancer Control TNM classification system, 8th edition.

^a^ The proportion mediated = indirect effect/(indirect effect + direct effect).

^b^ Decomposition of the total association between socioeconomic position (educational level, disposable income, and cohabiting status) and 5-year overall survival into pathways not via (direct effect) or via (indirect effect) the mediators (smoking status, comorbidity, clinical stage at diagnosis, and treatment intent).

^c^ Proportion mediated for clinical stage for patients with HPV-negative OPSCC with low compared with high disposable income was not applied because of opposite directions of the direct and indirect effects.

## Discussion

In this large, nationwide, population-based cohort study, we observed notable socioeconomic differences in 5-year survival probability among patients with a diagnosis of OPSCC. Although the subgroup of patients with HPV-positive OPSCC had a higher SEP and better survival than those with HPV-negative OPSCC, the socioeconomic gap in survival was remarkably similar in the 2 subgroups. Regardless of HPV status, patients with a lower educational level, with low disposable income, or living alone had a 10% to 15% lower overall survival probability at 5 years than patients with a higher educational level, high disposable income, or a cohabiting partner. Among patients with HPV-positive OPSCC, a considerable part (48%-75%) of the socioeconomic gap was estimated to be associated with differences in smoking (27%-48%), comorbidity (10%-19%), clinical stage at diagnosis (8%-19%), and treatment intent (16%-28%). Among patients with HPV-negative OPSCC, the combined proportion mediated by these factors was 24% to 60% and was associated mainly with differences in comorbidity (12%-22%) and treatment intent (16%-42%).

Previous studies^13,14,15,21,22,23,24^ may have underestimated the magnitude of the socioeconomic gap in OPSCC survival because the analyses were mutually adjusted for multiple socioeconomic and mediating factors,^14,15,21,22,23,24^ challenged by misclassification due to area-based socioeconomic parameters,^13,22^ or based on small cohorts (<200)^21,22,24^ (eFigure 4 in Supplement 1). These shortcomings may explain the findings of no statistically significant associations of income,^21,22^ insurance status,^23,24^ educational level,^21,22^ or marital status^23,24^ with survival after OPSCC by HPV status. However, 2 studies reported significantly higher hazard ratios for patients with HPV-positive OPSCC and/or those with HPV-negative OPSCC with low compared with high SEP, albeit the estimates were adjusted simultaneously for SEP indicators and mediating factors (eFigure 4 in Supplement 1).^14,15^ All but one^23^ of the previous studies had overall survival as the end point.^13,14,15,21,22,24^ The present study considers overall survival to address the total burden of socioeconomic differences in survival among patients with a diagnosis of OPSCC. Although this end point encompasses the general lower life expectancy among persons with low compared with high SEP, information on causes of death is subject to misclassification, which may be differential according to the patients’ SEP. Only 1 previous study approximated the interpretation of the extent to which prognostic factors explained the observed differences, by using a traditional mediation approach. This Canadian study by Chu et al^13^ (508 patients with HPV-positive OPSCC and 324 patients with HPV-negative OPSCC) observed no reduction in the hazard ratios when adding clinical stage (TNM-7) to a confounder-adjusted model; however, the hazard ratios diminished considerably when further adding smoking and alcohol consumption. This finding is in line with our results. In addition, we observed that TNM-8, which resulted in a down-staging of HPV-positive tumors,^41^ explained 8% to 19% of the observed socioeconomic gap in survival among patients with HPV-positive OPSCC. In prior research, the extent to which clinical stage at diagnosis explained socioeconomic differences in cancer survival seemed to vary across cancer sites and subsites.^9^ In regard to smoking, a study from the US (n = 23 923)^42^ and a study from the UK (n = 3440)^43^ including other HNSCC sites also found that differences in smoking behavior explained a considerable part of the socioeconomic gap in survival. We observed that the proportion of the socioeconomic gap explained by smoking seemed to be more extensive among patients with HPV-positive OPSCC than among those with HPV-negative OPSCC.

Patients with low SEP had more comorbidities. Regardless of HPV status, differences in comorbidities explained 10% to 22% of the observed socioeconomic gap in survival after OPSCC. To our knowledge, this finding has not previously been investigated but is in line with the findings in a study of patients with other HNSCC sites.^43^

In our analyses, we emphasized measuring characteristics at the first presentation at the oncologic center to elucidate the extent to which factors preceding the initiation of treatment explained the observed socioeconomic gap in OPSCC. Treatment intent may be considered to reflect a combined evaluation of the patient’s disease stage, health conditions, performance status, and capability to manage treatment. Treatment intent was associated with both SEP and survival and was estimated to explain 16% to 42% of the observed socioeconomic differences in survival. Although some previous studies included treatment intent in their analyses,^14,15,23,24^ the extent to which treatment intent explained the observed differences in survival cannot be elucidated from the reported estimates. This applies to most studies of other cancer sites,^9^ highlighting a gap in the literature.

### Strengths and Limitations

This study has some strengths. It is uniquely based on a Danish nationwide, population-based cohort of all patients with a diagnosis of OPSCC and treated according to standardized national guidelines. The linkage of prospectively registered information from a nationwide clinical database (DAHANCA) and administrative registries minimizes selection bias and ensures close to complete follow-up. Furthermore, this design allows for the examination of different aspects of SEP at an individual level, which reduces misclassification compared with area-based indicators of SEP. Another strength of the present study is its use of a counterfactual approach and effect estimates on the probability scale, instead of the hazard ratio scale, which offers a more direct interpretation.

This study also has some limitations. Socioeconomic position is a broad, multifaceted concept, which we approached by including 3 different proxy indicators: educational level, disposable income, and cohabitation status. The observed trends were largely similar, despite moderate variations in the estimated socioeconomic gap and indirect effects. However, the complex mechanisms between SEP and OPSCC survival and intercorrelated mediators (eFigure 2 in Supplement 1) limit causal interpretation, and our estimates could be biased owing to residual confounding of the exposure-mediator association or the mediator-outcome association. For example, because smokers and nonsmokers likely differ on other factors regarding the association with survival after OPSCC, the potential reduction by specifically eliminating socioeconomic differences in smoking behavior is likely smaller than the estimated indirect effects. On the other hand, the estimated indirect effects may be underestimated because of residual mediation. More detailed information on, for example, smoking behavior (eg, pack-years) would have enhanced our analyses, but this information is subject to missing values, and high generalizability is of major importance in studies exploring this hypothesis. Of the included patients, we excluded 12% owing to missing values, which is a substantially lower percentage than seen in most previous studies providing this information^13,14,15,22^ (eFigure 4 in Supplement 1). Despite the nationwide design, we still had few cases and wide 95% CIs in some strata, which limits firm conclusions.

In addition to potential residual mediation, the remaining gap may also be associated with differences in other patient characteristics at the time of diagnosis. Differences in alcohol consumption and performance status appeared particularly relevant; however, we did not have sufficient information to investigate these factors.

## Conclusions

Regardless of HPV status, patients with OPSCC with low SEP had 10% to 15% lower 5-year overall survival probability than patients with high SEP. A substantial part of this socioeconomic survival gap was estimated to be associated with smoking status, comorbidity, clinical stage, and treatment intent. This suggests that the observed socioeconomic differences in survival after OPSCC are, to a large degree, associated with differences in patient and disease characteristics at the time of diagnosis. This finding underscores the need for structural primary prevention initiatives targeting the systematic socioeconomic differences in health behavior and general health. Furthermore, our results highlight a large potential for tools that may identify vulnerable patients and for targeted interventions to support the best possible treatment and supportive care for this group.

## Notes

Supplement 1. **eTable 1.** Definitions of Main Variables **eTable 2.** Descriptive Characteristics of Patients Included (n = 4,053) and Excluded (n = 547) From the Analyzed Cohort **eTable 3.** Adjusted Odds Ratios (ORs) With 95% CIs for the Association Between Socioeconomic Position and Mediators **eTable 4.** Subanalyses of eTable 3 for TNM-7 Clinical Stage **eTable 5.** Estimated Direct and Indirect Effects With 95% CIs, the Mediator-Adjusted Model Includes Exposure-Mediator Interaction Term **eTable 6**. Hazard Ratios (HRs) for All-Cause Five-Year Mortality With 95% CIs According to Educational Level, Income and Cohabitation Status Among Patients With OPSCC, Denmark 2008-2019, Followed Through 2021 **eFigure 1.** Inclusion and Exclusion of Patients in the Study **eFigure 2.** Hypothesized Associations Between Socioeconomic Position and Survival After OPSCC Survival **eFigure 3.** Adjusted Hazard Ratios (HRs) for All-Cause Five-Year Mortality With 95% CIs for Low Versus High Socioeconomic Position **eFigure 4.** Summary of the Results of Previous Studies of Socioeconomic Position and Survival After Oropharyngeal Cancer According to or Adjusted by HPV Status
eMethods.

Supplement 2.
Data Sharing Statement
