# Supplementary material for: Association of Smoking, Comorbidity, Clinical Stage, and Treatment Intent With Socioeconomic Differences in Survival After Oropharyngeal Squamous Cell Carcinoma in Denmark
Source: JAMA Netw Open. 2022 Dec 7;5(12):e2245510. doi: 10.1001/jamanetworkopen.2022.45510 (PMC9856247; doi:10.1001/jamanetworkopen.2022.45510)
Supplement: Supplement 1. — eTable 1. Definitions of Main Variables eTable 2. Descriptive Characteristics of Patients Included (n = 4,053) and Excluded (n = 547) From the Analyzed Cohort eTable 3. Adjusted Odds Ratios (ORs) With 95% CIs for the Association Between Socioeconomic Position and Mediators eTable 4. Subanalyses of eTable 3 for TNM-7 Clinical Stage eTable 5. Estimated Direct and Indirect Effects With 95% CIs, the Mediator-Adjusted Model Includes Exposure-Mediator Interaction Term eTable 6. Hazard Ratios (HRs) for All-Cause Five-Year Mortality With 95% CIs According to Educational Level, Income and Cohabitation Status Among Patients With OPSCC, Denmark 2008-2019, Followed Through 2021 eFigure 1. Inclusion and Exclusion of Patients in the Study eFigure 2. Hypothesized Associations Between Socioeconomic Position and Survival After OPSCC Survival eFigure 3. Adjusted Hazard Ratios (HRs) for All-Cause Five-Year Mortality With 95% CIs for Low Versus High Socioeconomic Position eFigure 4. Summary of the Results of Previous Studies of Socioeconomic Position and Survival After Oropharyngeal Cancer According to or Adjusted by HPV Status eMethods. [file jamanetwopen-e2245510-s001.pdf]

Supplementary Online Content

Olsen MH, Frederiksen K, Lassen P, et al; Danish Head and Neck Cancer Group (DAHANCA). Association of smoking, comorbidity, clinical stage, and treatment intent with socioeconomic differences in survival after oropharyngeal squamous cell carcinoma in Denmark. *JAMA Netw Open.* 2022;5(12):e2245510.
doi:10.1001/jamanetworkopen.2022.45510

eTable 1. Definitions of Main Variables
eTable 2. Descriptive Characteristics of Patients Included (n = 4,053) and Excluded (n = 547) From the Analyzed Cohort
eTable 3. Adjusted Odds Ratios (ORs) With 95% CIs for the Association Between Socioeconomic Position and Mediators
eTable 4. Subanalyses of eTable3 for TNM-7 Clinical Stage
eTable 5. Estimated Direct and Indirect Effects With 95% CIs, the Mediator-Adjusted Model Includes Exposure-Mediator Interaction Term
eTable 6. Hazard Ratios (HRs) for All-Cause Five-Year Mortality With 95% CIs According to Educational Level, Income and Cohabitation Status Among Patients With OPSCC, Denmark 2008-2019, Followed Through 2021
eFigure 1. Inclusion and Exclusion of Patients in the Study
eFigure 2. Hypothesized Associations Between Socioeconomic Position and Survival After OPSCC Survival
eFigure 3. Adjusted Hazard Ratios (HRs) for All-Cause Five-Year Mortality With 95% CIs for Low Vs High Socioeconomic Position
eFigure 4. Summary of the Results of Previous Studies of Socioeconomic Position and Survival After Oropharyngeal Cancer According to or Adjusted by HPV Status
eMethods.

This supplementary material has been provided by the authors to give readers additional information about their work.

© 2022 Olsen MH et al. *JAMA Network Open.*

eTable 1. Definitions of Main Variables

**Socioeconomic indicators**	
**Educational level**<br>Lower<br>Medium<br>Higher	Highest attained educational level as registered in the year prior to the diagnosis. Lower education includes mandatory primary education (and lower secondary education for patients born ⩾ 1958), medium education includes upper secondary or vocational education (or lower secondary education for patients born <1958), and higher education includes higher education. The categorization differentiates according to birth year because of changes in the Danish schooling system.<br>Information on educational level is not available for patients born before 1921, and patients below age 30 are likely still undergoing education and thus may be considered not to have established their socioeconomic position yet. Accordingly, only patients born ⩾1921 and age ⩾30 were included.
**Disposable income**<br>Low<br>Medium<br>High	Disposable personal income for the calendar year before diagnosis. Calculated into quintiles according to that of the entire Danish population with the same legal gender and birth year. Categorized as low (1st quintile), medium (2nd and 3rd quintile), or high (4th and 5th quintile).
**Cohabitation status**<br>Living alone<br>Cohabiting	Cohabiting status by 1 January of the year of diagnosis. Cohabiting was defined as married, registered partner, or residing at the same address with a person: of the opposite legal gender, above age 16 years, and with an age difference of ≤15 years, with no kinship relations, and with no other adults, except for own children, in residence.
**Mediators**	
**Smoking**<br>Current smoker<br>Former/never-smoker	Patient-reported smoking status at the time of diagnosis. Former/never-smoker included recent and long-term quitters and patients with no history of smoking.
**Comorbidity**<br>HN-CCI ≥1<br>HN-CCI = 0	Comorbidity was based on the revised Charlson Comorbidity Index for Head and Neck Cancer (HN-CCI). A HN-CCI ≥ 1 was defined as hospitalization or outpatient visit in the period between 10 years and 30 days prior to HNSCC diagnosis with: congestive heart failure, cerebrovascular disease, chronic pulmonary disease, gastric ulcer disease, liver disease or diabetes, as registered in the Danish National Patient Register.
**Clinical stage (TNM-8)**<br>Advanced<br>Early	Clinical stage was measured at the time of diagnosis and defined according to the UICC TNM- classification 8th edition or, in subanalysis, according to the UICC TNM- classification 7th edition. Clinical stage was categorized as early stage (TNM: I-II), and advanced stage (TNM: III-IV).
**Treatment intent**<br>Palliative or no treatment<br>Curative	Treatment intent at the time of diagnosis was defined as curative intent vs palliative treatment or no treatment according to the DAHANCA national clinical guidelines.

HN-CCI = Revised Charlson Comorbidity Index for Head and Neck Cancer
DAHANCA = Danish Head And Neck Cancer Group

© 2022 Olsen MH et al. *JAMA Network Open.*

eTable 2. Descriptive Characteristics of Patients Included (n = 4,053) and Excluded (n = 547) From the Analyzed Cohort

Characteristics	Included (n = 4,053) No. (%)	Excluded (n = 547) No. (%)	P-value
Age, y			
30-53	785 (19)	94 (17)	P < 0.001
54-58	781 (19)	86 (16)	
59-63	826 (20)	109 (20)	
64-68	760 (19)	92 (17)	
69-95	901 (22)	166 (30)	
Median (IQR)	61 (55;68)	62 (56;70)	
Gender			
Male	3008 (74)	412 (75)	p = 0.56
Female	1045 (26)	135 (25)	
Year of diagnosis			
2008-2010	699 (17)	191 (35)	p < 0.001
2011-2013	1012 (25)	124 (23)	
2014-2016	1137 (28)	115 (21)	
2017-2019	1205 (30)	117 (21)	
Educational level (missing educational level or cohabitation status n = 93)			
Lower	857 (21)	112 (25)	p < 0.001
Medium	2336 (58)	268 (59)	
Higher	860 (21)	74 (16)	
Disposable income			
Low	1002 (25)	145 (27)	p = 0.59
Medium	1727 (43)	235 (43)	
High	1324 (33)	167 (31)	
Cohabitation status (missing educational level or cohabitation status n = 93)			
Living alone	1636 (40)	218 (48)	p < 0.001
Cohabiting	2417 (60)	236 (52)	
HPV status (missing n = 301)			
Positive	2563 (63)	139 (57)	p < 0.001
Negative	1490 (37)	107 (43)	
Smoking status (missing n = 185)			
Current smoker	1611 (40)	184 (51)	p < 0.001
Former/never-smoker	2442 (60)	178 (49)	
Comorbidities according to HN-CCI			
HN-CCI ≥ 1	989 (24)	191 (35)	p < 0.001
HN-CCI = 0	3064 (76)	356 (65)	
Clinical stage (TNM-8) (missing n = 19)			
Advanced	1472 (36)	338 (64)	p < 0.001
Early	2581 (64)	190 (36)	
Treatment intent			
Palliative or no treatment	351 (9)	113 (21)	p < 0.001
Curative	3702 (91)	434 (79)	

Abbrivations: HPV, human papillomavirus; HN-CCI, Revised Charlson Comorbidity Index for Head and Neck Cancer; TNM-8, American Joint Committee on Cancer/Union for International Cancer Control TNM classification system, 8th edition.

© 2022 Olsen MH et al. *JAMA Network Open.*

eTable 3. Adjusted Odds Ratios (ORs) With 95% CIs for the Association Between Socioeconomic Position and Mediators

	HPV-positive OPSCC				HPV-negative OPSCC			
	**Smoking[a]**	**Comorbidity[b]**	**Clinical stage[c]**	**Treatment intent[d]**	**Smoking[a]**	**Comorbidity[b]**	**Clinical stage[c]**	**Treatment intent[d]**
Indicator	OR[e] (95% CI)	OR[e] (95% CI)	OR[e] (95% CI)	OR[e] (95% CI)	OR[e] (95% CI)	OR[e] (95% CI)	OR[e] (95% CI)	OR[e] (95% CI)
Educational level								
Lower	3.5 (2.6 to 4.8)	2.5 (1.8 to 3.5)	1.7 (1.2 to 2.4)	10.1 (3.9 to 26.2)	1.7 (1.2 to 2.5)	1.7 (1.2 to 2.5)	1.3 (0.9 to 2.0)	2.0 (1.2 to 3.2)
Medium	2.3 (1.8 to 3.0)	1.7 (1.3 to 2.2)	1.1 (0.8 to 1.5)	6.0 (2.4 to 15.2)	1.5 (1.1 to 2.0)	1.5 (1.0 to 2.0)	1.2 (0.8 to 1.6)	1.5 (0.9 to 2.3)
Higher	1.0 [Reference]	1.0 [Reference]	1.0 [Reference]	1.0 [Reference]	1.0 [Reference]	1.0 [Reference]	1.0 [Reference]	1.0 [Reference]
Disposable income								
Low	4.2 (3.3 to 5.5)	2.3 (1.7 to 3.0)	1.7 (1.3 to 2.4)	2.6 (1.5 to 4.5)	1.4 (1.0 to 2.0)	1.8 (1.3 to 2.5)	0.9 (0.7 to 1.4)	1.5 (0.9 to 2.2)
Medium	2.4 (1.9 to 3.0)	1.7 (1.3 to 2.1)	1.0 (0.8 to 1.3)	1.8 (1.1 to 3.0)	1.3 (1.0 to 1.8)	1.7 (1.2 to 2.2)	0.9 (0.6 to 1.3)	1.5 (1.0 to 2.2)
High	1.0 [Reference]	1.0 [Reference]	1.0 [Reference]	1.0 [Reference]	1.0 [Reference]	1.0 [Reference]	1.0 [Reference]	1.0 [Reference]
Cohabitation status								
Living alone	2.6 (2.1 to 3.1)	1.7 (1.4 to 2.1)	1.3 (1.0 to 1.7)	2.4 (1.6 to 3.6)	1.9 (1.5 to 2.4)	1.5 (1.2 to 1.8)	1.1 (0.9 to 1.5)	2.4 (1.7 to 3.2)
Cohabiting	1.0 [Reference]	1.0 [Reference]	1.0 [Reference]	1.0 [Reference]	1.0 [Reference]	1.0 [Reference]	1.0 [Reference]	1.0 [Reference]

Abbrivations: HPV, human papillomavirus; OPSCC, oropharyngeal squamous cell carcinoma; HN-CCI, Revised Charlson Comorbidity Index for Head and Neck Cancer
[a] OR for current vs former or never-smoker
[b] OR for HN-CCI ≥ 1 vs HN-CCI = 0
[c] OR for advanced vs early TNM-8 clinical stage
[d] OR for palliative or no treatment vs curative treatment intent
[e]Adjusted for gender, age (continous), and calendar year (continous) at diagnosis

© 2022 Olsen MH et al. *JAMA Network Open.*

eTable 4. Subanalyses of eTable3 for TNM-7 Clinical Stage

	**HPV positive OPSCC**	**HPV negative OPSCC**
	**TNM-7 Clinical stage**	**TNM-7 Clinical stage**
Indicator	OR[a] (95% CI)	OR[a] (95% CI)
Educational level		
Lower	1.1 (0.7 to 1.7)	1.3 (0.9 to 2.0)
Medium	1.0 (0.7 to 1.4)	1.2 (0.8 to 1.7)
Higher	1.0 [Reference]	1.0 [Reference]
Disposable income		
Low	0.7 (0.5 to 0.9)	0.9 (0.6 to 1.3)
Medium	1.0 (0.7 to 1.3)	0.9 (0.6 to 1.2)
High	1.0 [Reference]	1.0 [Reference]
Cohabitation status		
Living alone	1.4 (1.0 to 1.8)	1.1 (0.9 to 1.4)
Cohabiting	1.0 [Reference]	1.0 [Reference]

Abbrivations: HPV, human papillomavirus; OPSCC, oropharyngeal squamous cell carcinoma;
HN-CCI, Revised Charlson Comorbidity Index for Head and Neck Cancer
[a] OR for advanced vs early TNM-7 clinical stage adjusted for gender, age (continous), and calendar year (continous) at diagnosis

© 2022 Olsen MH et al. *JAMA Network Open.*

eTable 5. Estimated Direct and Indirect Effects With 95% CIs, the Mediator-Adjusted Model Includes Exposure-Mediator Interaction Term

	**HPV-positive OPSCC**			**HPV-negative OPSCC**		
	**Survival difference % (95% CI)**		**Proportion mediated, %[b]**	**Survival difference % (95% CI)**		**Proportion mediated, %[b]**
**Indicator**	**Direct effect[a]**	**Indirect effect[a]**		**Direct effect[a]**	**Indirect effect[a]**	
Educational level (Lower vs higher)						
Smoking status	-10.1 (-15.0 to -5.3)	-5.2 (-7.9 to -3.2)	34.2	-13.8 (-21.4 to -5.7)	-1.0 (-2.8 to 0.0)	6.8
Comorbidity (HN-CCI)	-13.1 (-17.6 to -8.3)	-2.3 (-4.2 to -0.9)	15.1	-11.7 (-19.2 to -3.1)	-2.6 (-5.1 to -0.9)	17.9
Clinical stage (TNM-8)	-13.2 (-18.4 to -8.8)	-1.6 (-3.2 to -0.4)	10.8	-12.9 (-20.4 to -5.2)	-1.5 (-4.1 to 0.6)	10.4
Treatment intent	-11.6 (-16.0 to -7.8)	-3.6 (-5.3 to -2.3)	23.7	-11.4 (-19.1 to -3.8)	-2.9 (-5.1 to -0.9)	20.3
Disposable income (Low vs high)						
Smoking status	-4.1 (-8.4 to 0.2)	-7.6 (-10.3 to -5.0)	65.0	-9.1 (-15.9 to -2.7)	-0.5 (-1.6 to 0.1)	5.6
Comorbidity (HN-CCI)	-10.0 (-14.6 to -5.6)	-1.7 (-3.5 to -0.6)	14.9	-8.0 (-15.0 to -1.6)	-1.8 (-3.3 to -0.5)	18.4
Clinical stage (TNM-8)	-9.6 (-14.0 to -5.4)	-2.0 (-3.5 to -0.7)	17.3	-9.8 (-16.4 to -4.1)	0.4 (-2.0 to 2.7)	NA
Treatment intent	-10.2 (-14.9 to -6.0)	-1.9 (-3.4 to -0.6)	15.4	-6.3 (-12.8 to 0.0)	-1.7 (-3.7 to 0.3)	21.8
Cohabitation status (Living alone vs cohabiting)						
Smoking status	-10.6 (-14.3 to -7.2)	-3.9 (-5.4 to -2.5)	26.9	-10.7 (-15.4 to -6.0)	-1.0 (-2.3 to -0.1)	8.9
Comorbidity (HN-CCI)	-13.2 (-16.6 to -9.7)	-1.4 (-2.5 to -0.6)	9.7	-10.8 (-14.9 to -6.0)	-1.1 (-2.0 to -0.4)	9.1
Clinical stage (TNM-8)	-13.8 (-17.3 to -10.1)	-0.8 (-1.8 to 0.0)	5.8	-11.2 (-15.6 to -6.2)	-0.5 (-1.8 to 0.6)	4.4
Treatment intent	-13.2 (-16.8 to -9.2)	-1.8 (-3.0 to -0.8)	12.0	-7.5 (-12.1 to -2.8)	-3.9 (-5.4 to -2.6)	34.4

Abbrivations: HPV, human papillomavirus; OPSCC, oropharyngeal squamous cell carcinoma; HN-CCI, Revised Charlson Comorbidity Index for Head and Neck Cancer
NA: Proportion mediated for clinical stage for patients with HPV- OPSCC with low compared to high income was not applied because of inconsistencies in the direction of the indirect effects. Further, the combined associations of smoking, comorbidity, clinical stage, and treatment intent could not be estimated in these models including an exposure-mediator interaction-term

[a] Decomposition of the associations between socioeconomic position (education, income, or cohabiting status) and five-year overall survival into pathways not via (direct effects) or via (indirect effects) the mediators (smoking status, comorbidity, clinical stage at diagnosis, or/and treatment intent).

[b] The indirect effect/(indirect effect +direct effect).

© 2022 Olsen MH et al. *JAMA Network Open.*

eTable 6. Hazard Ratios (HRs) for All-Cause Five-Year Mortality With 95% CIs According to Educational Level, Income and Cohabitation Status Among Patients With OPSCC, Denmark 2008-2019, Followed Through 2021

	**HPV-positive OPSCC**				**HPV-negative OPSCC**			
	**Analyzed cohort**	**Missing HPV status = HPV+**	**Missing education = lower**	**Missing smoking = current smoker**	**Analyzed cohort**	**Missing HPV status = HPV-**	**Missing education = lower**	**Missing smoking = current smoker**
	**(n = 2,563)**	**(n = 2,830)**	**(n = 2,613)**	**(n = 2,648)**	**(n = 1,490)**	**(n = 1,757)**	**(n = 1,526)**	**(n = 1,549)**
**Indicator**	**HR[a] (95% CI)**	**HR[a] (95% CI)**	**HR[a] (95% CI)**	**HR[a] (95% CI)**	**HR[a] (95% CI)**	**HR[a] (95% CI)**	**HR[a] (95% CI)**	**HR[a] (95% CI)**
Educational level								
Lower	2.3 (1.8 to 3.0)	2.6 (2.0 to 3.3)	2.3 (1.7 to 3.0)	2.1 (1.6 to 2.8)	1.5 (1.2 to 1.9)	1.6 (1.3 to 2.0)	1.5 (1.2 to 1.9)	1.5 (1.2 to 1.9)
Medium	1.7 (1.4 to 2.2)	1.8 (1.4 to 2.3)	1.7 (1.4 to 2.2)	1.6 (1.3 to 2.0)	1.3 (1.1 to 1.6)	1.3 (1.1 to 1.6)	1.3 (1.1 to 1.6)	1.3 (1.1 to 1.6)
Higher	1.0 [Reference]	1.0 [Reference]	1.0 [Reference]	1.0 [Reference]	1.0 [Reference]	1.0 [Reference]	1.0 [Reference]	1.0 [Reference]
Disposable Income								
Low	1.8 (1.4 to 2.2)	1.9 (1.5 to 2.3)	1.8 (1.4 to 2.2)	1.9 (1.5 to 2.3)	1.3 (1.1 to 1.6)	1.5 (1.2 to 1.8)	1.3 (1.1 to 1.6)	1.3 (1.1 to 1.6)
Medium	1.2 (1.0 to 1.4)	1.3 (1.1 to 1.6)	1.2 (1.0 to 1.5)	1.2 (1.0 to 1.5)	1.2 (1.0 to 1.4)	1.3 (1.1 to 1.6)	1.2 (1.0 to 1.4)	1.2 (1.0 to 1.5)
High	1.0 [Reference]	1.0 [Reference]	1.0 [Reference]	1.0 [Reference]	1.0 [Reference]	1.0 [Reference]	1.0 [Reference]	1.0 [Reference]
Cohabitation status								
Living alone	2.1 (1.8 to 2.5)	2.2 (1.8 to 2.5)	2.1 (1.8 to 2.5)	2.0 (1.7 to 2.4)	1.4 (1.2 to 1.6)	1.5 (1.3 to 1.7)	1.4 (1.2 to 1.6)	1.4 (1.3 to 1.6)
Cohabiting	1.0 [Reference]	1.0 [Reference]	1.0 [Reference]	1.0 [Reference]	1.0 [Reference]	1.0 [Reference]	1.0 [Reference]	1.0 [Reference]

Abbrivations: HPV, human papillomavirus; OPSCC, oropharyngeal squamous cell carcinoma; HN-CCI, Revised Charlson Comorbidity Index for Head and Neck Cancer
[a]Adjusted for gender, age (continous) and calendar year (continous) at diagnosis

© 2022 Olsen MH et al. *JAMA Network Open.*

eFigure 1. Inclusion and Exclusion of Patients in the Study

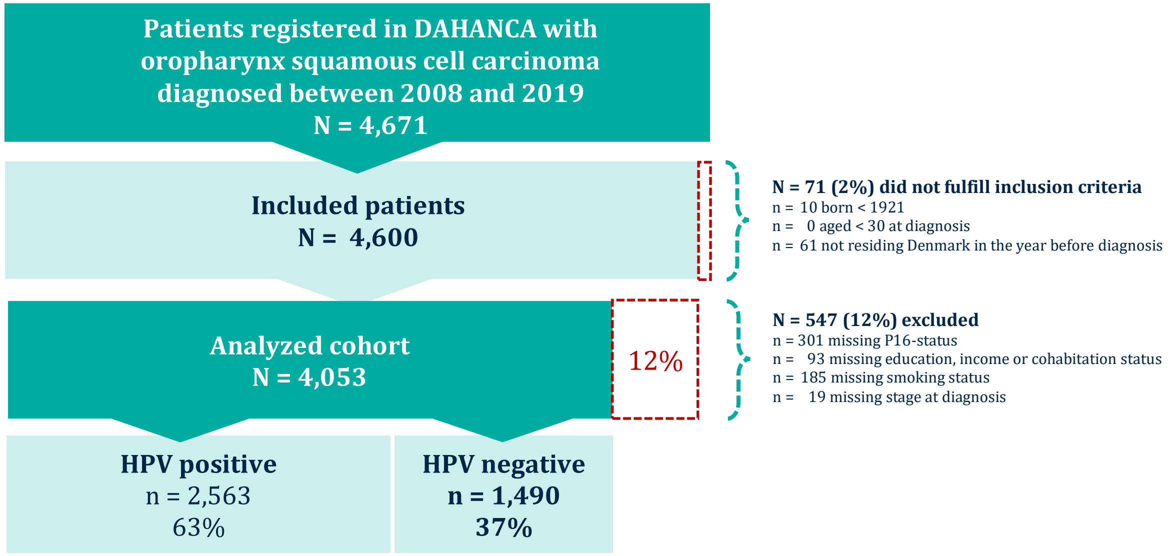

© 2022 Olsen MH et al. *JAMA Network Open.*

eFigure 2. Hypothesized Associations Between Socioeconomic Position and Survival After OPSCC Survival

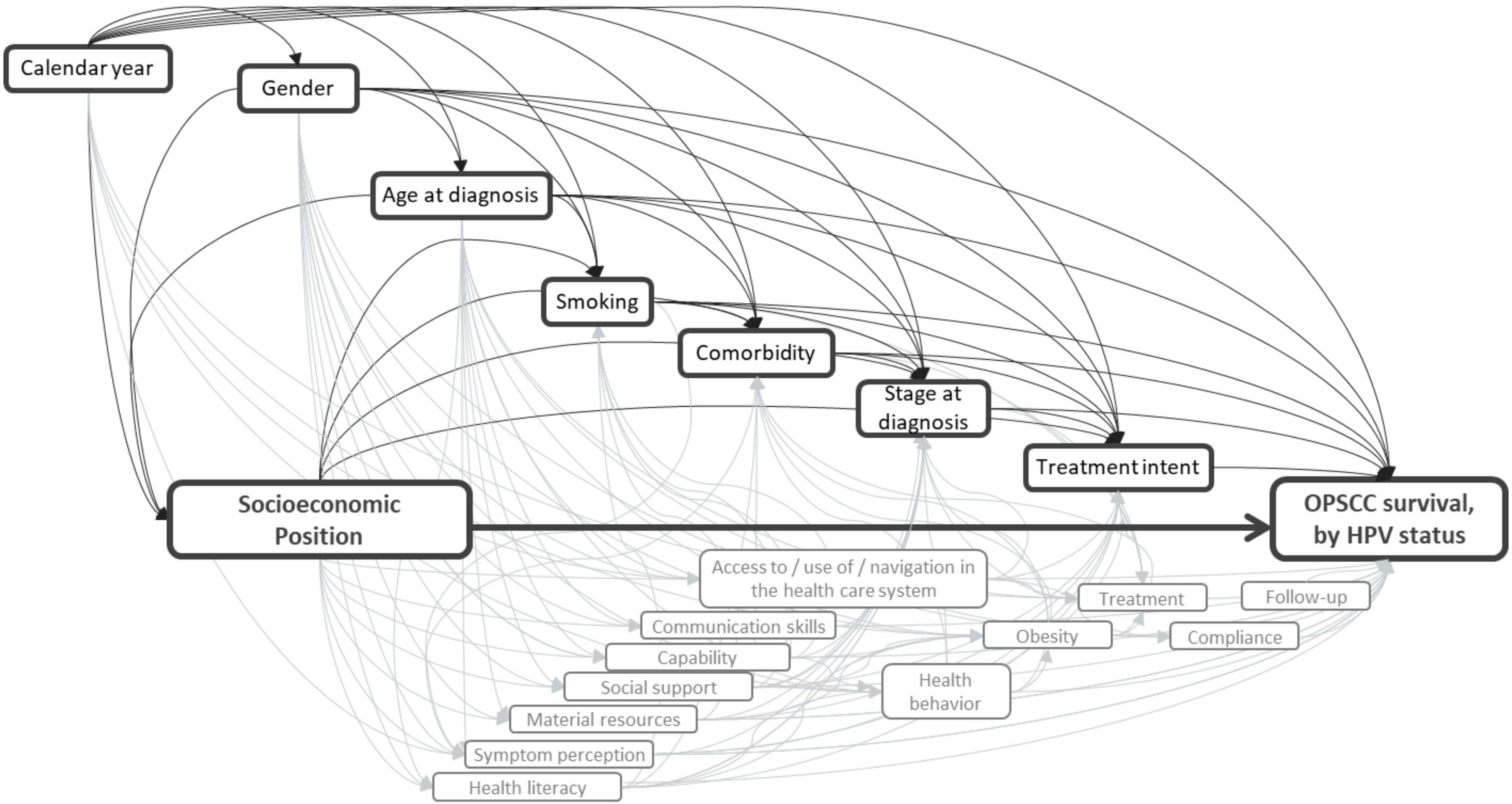

OPSCC = oropharyngeal squamous cell carcinoma
HPV = human papiloma virus
Hypothesized pathways: Socioeconomic position was measured by three proxy indicators: educational level, disposable income and cohabitation status. All three indicators are hypothesized to be associated with survival after oropharyngeal squamous cell carcinoma (OPSCC), by human papillomavirus (HPV) status, through numerous pathways. Structural and cultural differences as well as preceding factors related to and the abilities attained from education, are hypothesized to reflect factors such as: health literacy, symptom perception, health behavior, capability, communication skills, and use of and navigation in the health care system. Whereas educational level is considered as a stable indicator over time, disposable income changes considerably over time, and reflects to a higher extent the patients' current socioeconomic position in society as well as material resources. Finally, cohabitation status is hypothesized to reflect, e.g., social and practical support, health behavior, health care seeking behavior, and navigation in the health care system.

© 2022 Olsen MH et al. *JAMA Network Open.*

eFigure 3. Adjusted Hazard Ratios (HRs) for All-Cause Five-Year Mortality With 95% CIs for Low Vs High Socioeconomic Position

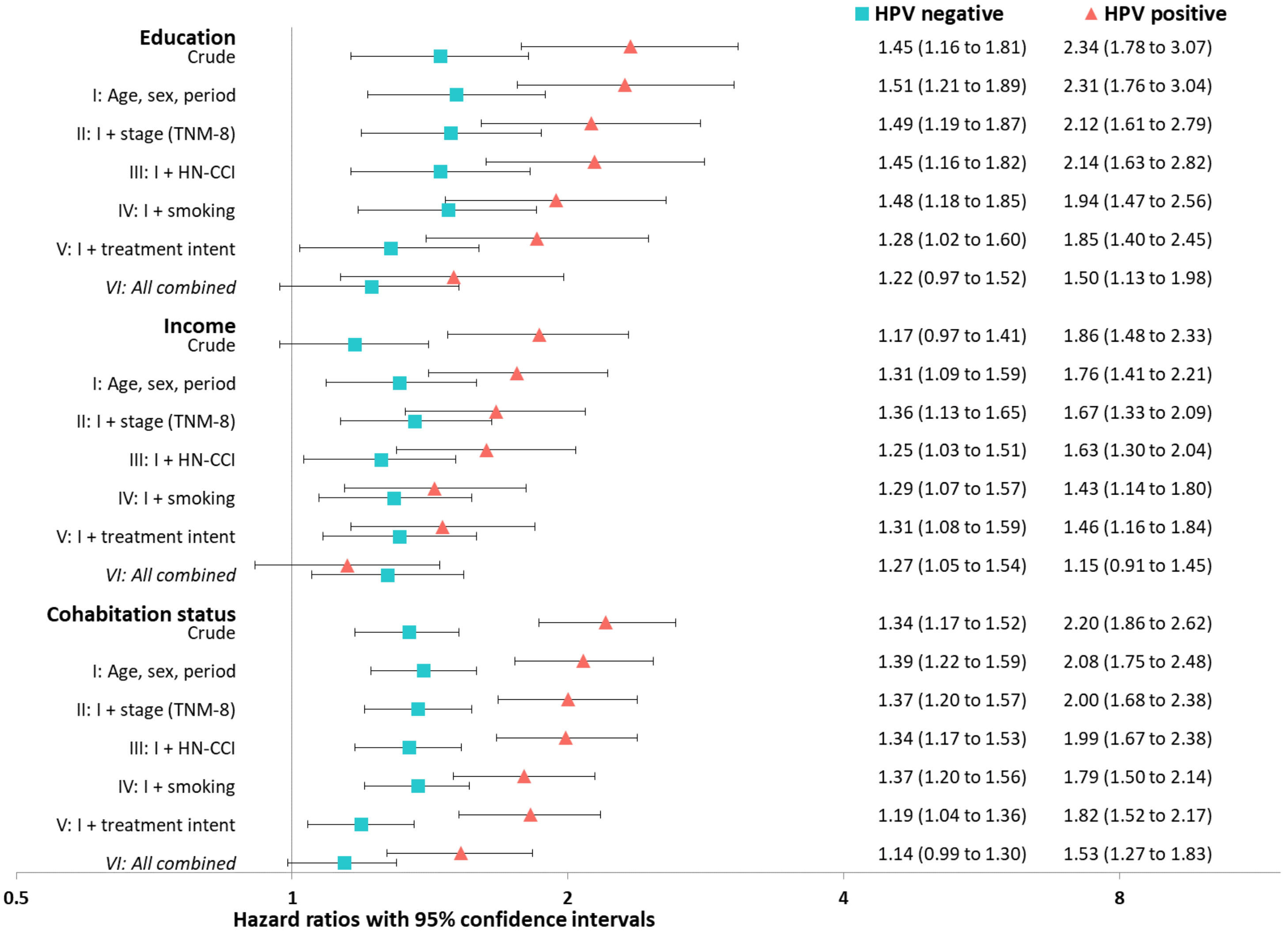

Abbrivations: HPV, human papillomavirus; HN-CCI, Revised Charlson Comorbidity Index for Head and Neck Cancer

Reference categories: Education: HR for lower education, reference: higher education, Income: HR for low income (1st quintile), reference high income (4th-5th quintile) Cohabitation status: HR for living alone, reference: cohabiting. **Adjustments**: I: The HRs are adjusted for age, gender, and year of diagnosis. II: HRs are adjusted for age, gender, year of diagnosis, and clinical stage at diagnosis, III: HRs are adjusted for age, gender, year of diagnosis, and comorbidity (HN-CCI), IV: HRs are adjusted for age, gender, year of diagnosis, and smoking, V: HRs are adjusted for age, gender, year of diagnosis, and treatment intent, VI: HRs are adjusted for age, gender, year of diagnosis, clinical stage, comorbidity (HN-CCI), smoking status, and treatment intent at diagnosis.

© 2022 Olsen MH et al. *JAMA Network Open.*

eFigure 4. Summary of the Results of Previous Studies of Socioeconomic Position and Survival After Oropharyngeal Cancer According to or Adjusted by HPV Status

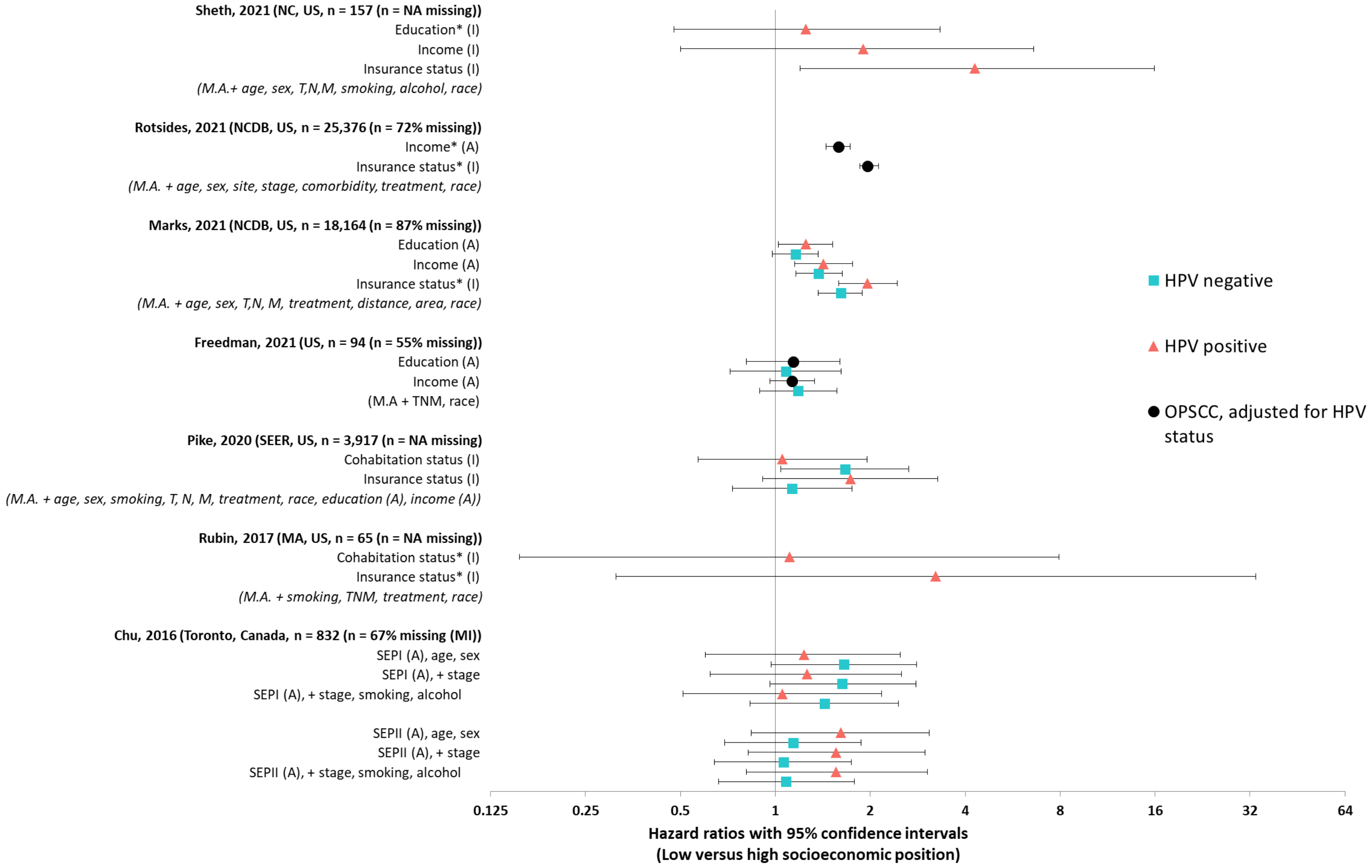

Abbrivations: HPV, human papillomavirus; OPSCC, oropharyngeal squamous cell carcinoma
M.A = the estimates are multiple adjusted by all variables listed, (I) = individual-level SEP indicator. (A) = Area-based SEP-indicator. MI = missing values are imputed. NA = missing values are not applied, * = The reference-group are converted (1/estimate) to illustrate trends.

© 2022 Olsen MH et al. *JAMA Network Open.*

eMethods.

The following expressions was used to estimate the Direct effect and Indirect effect:

$$Direct\ effect = \frac{1}{N}\sum_{i,m}\left[\hat{S}(\,5\ years|E = e^{*}, C = c_i, M = m) - \hat{S}(5\ years|E = e, C = c_i, M = m)\right] \cdot \hat{P}(M = m|E = e, C = c_i)$$

$$Indirect\ effect = \frac{1}{N}\sum_{i,m}\hat{S}(5\ years|E = e^{*}, C = c_i, M = m) \cdot [\hat{P}(M = m|\,E = e^{*}, C = c_i) - \hat{P}(M = m|E = e, C = c_i)]$$

with E denoting exposure and e^{*} denoting the exposed and e the reference level; C confounders and c_i confounder values for individual i i=1,…N; and M mediator. The survival probabilities $\widehat{S}$ were estimated from confounder-adjusted Cox regression model including the mediator, and using the Breslow method to estimate the baseline hazard. $\hat{P}$ was derived from the confounder-adjusted exposure-mediator logistic regression model.

© 2022 Olsen MH et al. *JAMA Network Open.*
