# Supplementary material for: Association of Smoking, Comorbidity, Clinical Stage, and Treatment Intent With Socioeconomic Differences in Survival After Oropharyngeal Squamous Cell Carcinoma in Denmark
Source: JAMA Netw Open. 2022 Dec 7;5(12):e2245510. doi: 10.1001/jamanetworkopen.2022.45510 (PMC9856247; doi:10.1001/jamanetworkopen.2022.45510)
Supplement: Supplement 2. — Data Sharing Statement [file jamanetwopen-e2245510-s002.pdf]

Data Sharing Statement

Olsen. Association of Smoking, Comorbidity, Clinical Stage, and Treatment Intent With Socioeconomic Differences in Survival After Oropharyngeal Squamous Cell Carcinoma in Denmark. *JAMA Netw Open*. Published December 07, 2022. doi:10.1001/jamanetworkopen.2022.45510

Data
Data available: No

Additional Information
Explanation for why data not available: All data were de-identified, linked and accessed through the Danish Cancer Society Research Center's secure server at Statistics Denmark. The authors do not have permission to share the data.
